# Podoplanin-expressing inflammatory macrophages activate murine platelets via CLEC-2

**DOI:** 10.1111/j.1538-7836.2011.04614.x

**Published:** 2012-03

**Authors:** A M Kerrigan, L Navarro-Nuñez, E Pyz, B A Finney, J A Willment, S P Watson, G D Brown

**Affiliations:** * Section of Immunology and Infection, Division of Applied Medicine, Institute of Medical Sciences, University of AberdeenAberdeen, UK; † Centre for Cardiovascular Sciences, Institute of Biomedical Research, College of Medical and Dental Sciences, University of BirminghamBirmingham, UK; ‡ Clinical Laboratory Sciences, Division of Immunology, Institute of Infectious Disease and Molecular Medicine, University of Cape TownCape Town, South Africa

Podoplanin, a small transmembrane glycoprotein, is expressed at high levels on lymphatic endothelial cells, kidney podocytes and lung type I alveolar cells, but absent on blood endothelial cells. It has also been described on splenic and peritoneal macrophages, but the functional significance of this is unknown [1]. Podoplanin is an endogenous ligand for CLEC-2, a C-type lectin expressed on platelets and at a lower level on subsets of other hematopoietic cells [2–4]. The interaction of CLEC-2 on platelets with podoplanin on lymphatic endothelial cells is essential for the separation of lymphatic and blood vessels [5,6]. In the present study, we further investigated the expression of podoplanin on macrophages and explored the ability of macrophage-expressed podoplanin to function as a CLEC-2 ligand.

**Fig. 1:**
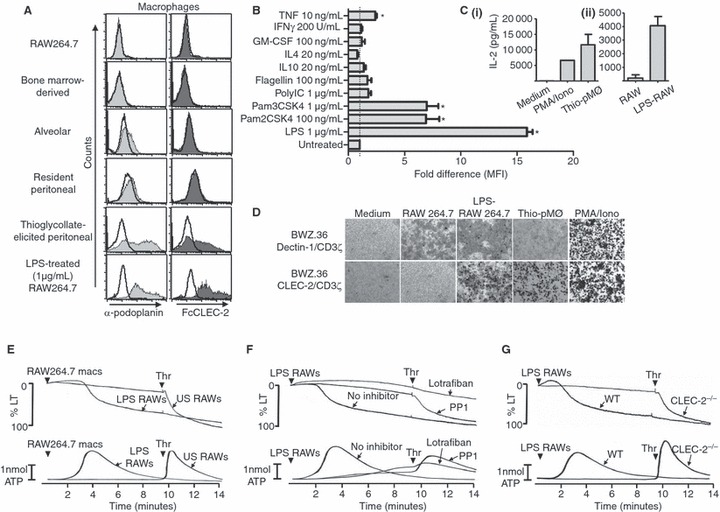
Podoplanin expression on macrophages. (A) Anti-podoplanin and FcCLEC-2 staining of various macrophage populations as indicated. Resident and thioglycollate-elicited peritoneal macrophages and resident alveolar macrophages were isolated from Balb/c mice by standard procedures. Bone marrow-derived macrophages (BMDMs) were generated from Balb/c mice by standard procedures. Filled histograms represent podoplanin staining (light grey) or FcCLEC-2 staining (dark grey). rIgG2a isotype control staining (left panel) or FcDectin-1 control staining (right panel) are represented by unfilled histograms. In all cases, detection of binding of the primary probe was with PE-conjugated secondary antibodies. Flow cytometry analysis was performed following excitation with 488 nm and detection with a 585 ± 42 nm band pass filter. Data are representative of at least three independent experiments. (B) The fold changes in podoplanin expression (represented by mean fluorescent intensities, MFI) by bone marrow-derived macrophages following stimulation for 20 h with various Toll-like receptor agonists and cytokines as indicated. Podoplanin expression was determined by flow cytometry as above. Data were generated from two independent experiments, each consisting of duplicate samples, normalized to the untreated control value and analyzed by one-way anova with Bonferroni post-tests to determine significant differences between untreated and treated cells. Error bars indicate the SEM. *P < 0.05 vs untreated control. (C) ELISA determination of interleukin (IL)-2 production from 1 × 10^5^ BWZ.36 CLEC-2/CD3ζ reporter cells (generated essentially as previously described [8]), following co-culture for 20 h with (i) thioglycollate-elicited macrophages (5 × 10^5^ to 1 × 10^6^) or PMA (65 nm) and ionomycin (2 μm) and (ii) unstimulated or LPS-treated (1 μg mL^−1^ for 20 h) RAW264.7 cells (1 × 10^5^ to 3 × 10^5^). The data were generated from two independent experiments. Error bars represent the SD. (D) X-gal staining using standard procedures of BWZ.36 CLEC-2/CD3ζ or Dectin-1/CD3ζ reporter cells following co-culture as before with various cell types as indicated, or stimulation with PMA/iomomycin as a positive control. Images are representative of at least three independent experiments (thioglycollate elicited peritoneal macrophages [Thio-pMØ]). (E) Unstimulated (US) or LPS-stimulated (1 μg mL^−1^ for 20 h) RAW264.7 macrophages (macs) were added to washed mouse platelet suspensions (3 × 10^8^ platelets per mL) at a macrophage:platelet ratio of 1:15. After 10 min, 0.1 U per mL thrombin (Thr) was added to assess the extent of the response elicited by the macrophages. (F) Washed platelets (3 × 10^8^ platelets per mL) in the absence or presence (arrows) of the Src family kinase inhibitor PP1 (10 μm) or the αIIbβ3 inhibitor lotrafiban (10 μm) were stimulated with LPS-activated RAW264.7 cells (macrophage:platelet ratio 1:15) for 10 min, followed by the addition of 0.1 U mL^−1^ thrombin. (G) CLEC-2-deficient washed platelets and platelets from litter-matched wild-type controls (3 × 10^8^ platelets per mL) were stimulated with LPS-activated RAW264.7 cells for 10 min, followed by the addition of 0.1 U per mL thrombin. Aggregation was quantified as a function of light transmission (LT), with 100% aggregation corresponding to 100% light transmission. Adenosine triphosphate (ATP) secretion was measured by the luciferase-luciferin method. Representative traces from three independent experiments shown. Arrowheads indicate agonist addition.

We probed various macrophage populations with a podoplanin-specific antibody and FcCLEC-2 (a probe for CLEC-2 ligands [7]). We were unable to detect specific binding of either the podoplanin antibody or FcCLEC-2 on RAW264.7 macrophages, bone marrow-derived macrophages (BMDMs) and tissue-resident macrophages, including alveolar and resident peritoneal macrophages, indicating the absence of podoplanin (Fig. 1A). Conversely, podoplanin was detected on thioglycollate-elicited ‘inflammatory’ peritoneal macrophages and lipopolysaccharide (LPS)-treated RAW264.7 cells using the same two ligands (Fig. 1A). Podoplanin expression was also markedly up-regulated on BMDMs following stimulation with LPS and weakly up-regulated in response to the TLR2/1 and TLR2/6 agonists, Pam3CSK4 and Pam2CSK4, respectively (Fig. 1B). There was also a slight, but significant, up-regulation induced by the inflammatory cytokine tumor necrosis factor (TNF), but not by several other cytokines tested (Fig. 1B). These results demonstrate that podoplanin is expressed on inflammatory but not tissue-resident macrophages, and is up-regulated in response to TLR stimulation and some inflammatory cytokines. The present results extend a previous report of podoplanin expression on macrophages [1], by revealing that expression is limited to inflammatory subsets.

To investigate whether macrophage-expressed podoplanin could induce CLEC-2 activation we used a reporter system based on BWZ.36 cells expressing a chimeric CLEC-2/CD3ζ receptor linked to β-galactosidase expression and IL-2 secretion [8]. Incubation of these cells with podoplanin-expressing macrophages induced IL-2 and β-galactosidase, whereas podoplanin-negative macrophages had no effect (Figs 1C and D). We also investigated whether podoplanin-expressing macrophages could directly induce platelet activation (determined by analysing aggregation and ATP secretion). Untreated podoplanin-negative RAW264.7 macrophages had no effect on platelet aggregation or ATP secretion, whereas the subsequent addition of thrombin induced powerful activation. In contrast, LPS-activated RAW264.7 macrophages stimulated platelet aggregation and ATP secretion after a delay of several minutes (Fig. 1E). A delay in activation is reminiscent of platelet activation by the CLEC-2 ligand rhodocytin [2]. The subsequent lack of an effect of addition of thrombin confirmed that these platelets had undergone full activation. Aggregation and secretion induced by LPS-stimulated RAW264.7 cells was blocked by the αIIbβ3 inhibitor, lotrafiban, confirming integrin-dependent platelet activation, and by the Src kinase inhibitor, PP1, consistent with CLEC-2-mediated platelet activation (Fig. 1F). In line with this, activation was blocked in CLEC-2-deficient mouse platelets [9], whereas thrombin was still able to induce full activation of these cells (Fig. 1G). Another mechanism by which platelets and macrophages interact is via binding of P-selectin on activated platelets to PSGL-1 on macrophages. We investigated whether blocking this interaction would modulate platelet activation triggered by podoplanin-expressing macrophages; however, the inclusion of a blocking antibody against PSGL-1 did not have any effect on platelet aggregation or secretion (data not shown).

These results demonstrate that inflammatory macrophages expressing podoplanin can directly activate platelets via CLEC-2. This could represent a novel mechanism for extravascular platelet activation during clotting, wound healing and vascular inflammatory processes such as atherosclerosis. Expression of podoplanin promotes migration of a variety of cell types, including tumor, MDCK and lymphatic endothelial cells [10–12]. The presence of podoplanin on macrophages could therefore underlie a mechanism through which platelets regulate migration of inflammatory macrophages as well as other functions.
